# Museum Specimens Reveal the Taxonomic Distinctions Among South Asian Treeshrews

**DOI:** 10.1002/ece3.71202

**Published:** 2025-04-23

**Authors:** Manokaran Kamalakannan, Mukesh Thakur, Nithyanandam Marimuthu, Subhojit Pramanik, Dhriti Banerjee

**Affiliations:** ^1^ Mammal and Osteology Section Zoological Survey of India, Ministry of Environment, Forest & Climate Change, Govt. of India Kolkata India; ^2^ Zoological Survey of India, Ministry of Environment, Forest & Climate Change, Govt. of India Kolkata India

**Keywords:** cranial‐morphology, endemic, monotypic, museum‐specimens, taxonomy, treeshrew

## Abstract

South Asian treeshrews include the Madras Treeshrew *Anathana ellioti* , the Northern Treeshrew *Tupaia belangeri* , and the Nicobar Treeshrew *Tupaia nicobarica* , each occupying distinct and non‐overlapping geographical areas in India and Southeast Asia. In this study, we investigated the morphological variation among these species using museum specimens collected over a wide spatial and temporal range of India and Myanmar and combined with existing published datasets. We analyzed 22 cranial measurements and four external traits to evaluate inter‐ and intraspecific morphological differentiation, employing distance‐based morphometric approaches validated by multivariate analyses. Our findings revealed considerable heterogeneity in cranial morphology, with three species exhibiting clear differentiation, despite slight overlaps in morphospace. Furthermore, our results support the synonymy of the five previously recognized allopatric subspecies of *T. belangeri* and the two subspecies of *A. ellioti* . The additional diagnostic characteristics identified in this study enhance the understanding of morphological distinctions among the South Asian treeshrews and contribute to broader taxonomic knowledge of treeshrew diversity.

## Graphical abstract

In this study, we investigated the morphological relationships among the treeshrew species found in South Asia using museum specimens collected over a wide spatial and temporal range in India and Myanmar, combined with existing published datasets, contributing to broader taxonomic knowledge of treeshrew diversity.

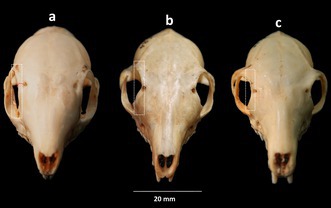

## 1 Introduction

**FIGURE 1:**
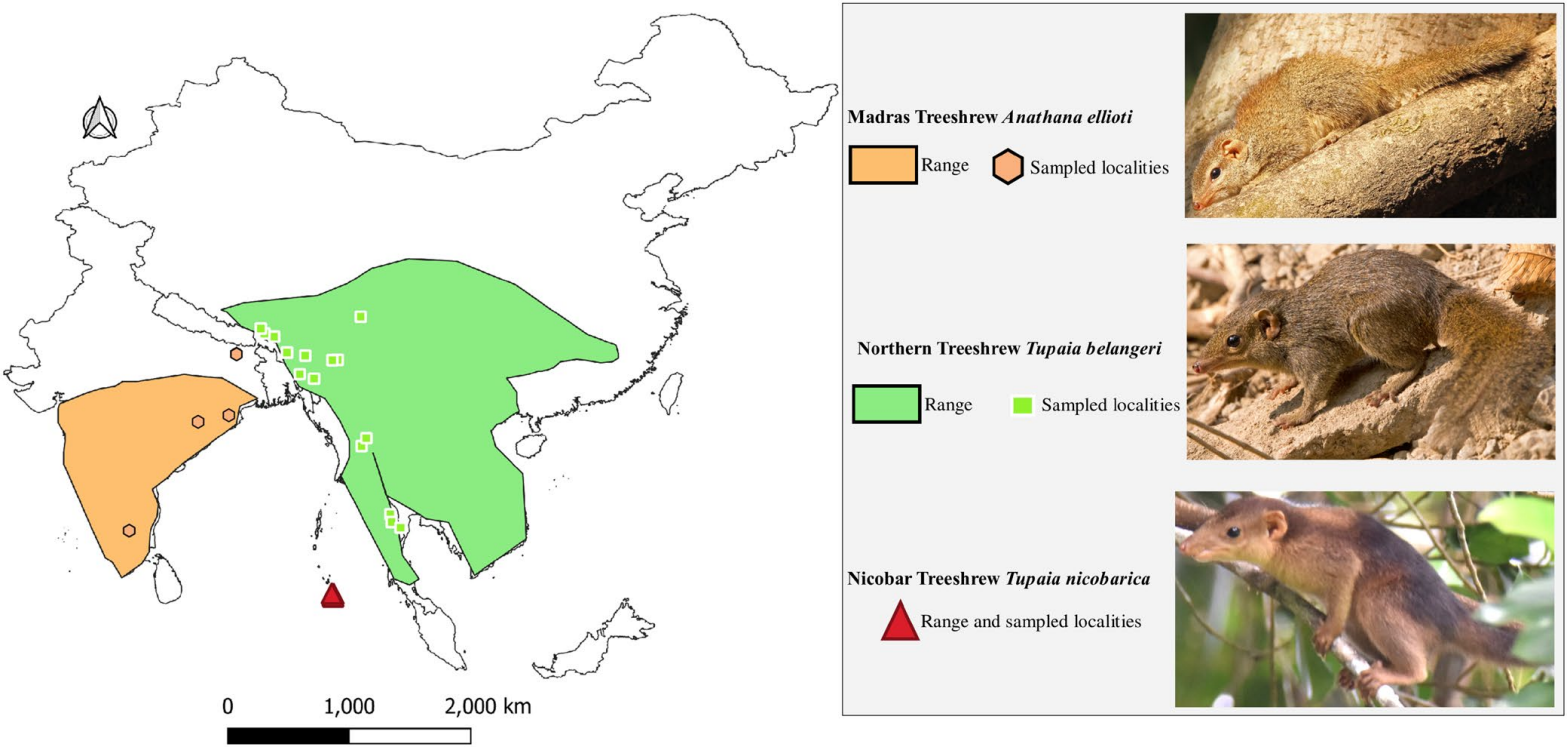
Map of South and Southeast Asia showing the range of South Asian treeshrews ( *Anathana ellioti* , *Tupaia belangeri* and *Tupaia nicobarica* ) along with the localities of museum specimens. The global distribution of South Asian treeshrews is based on the IUCN Red List of Threatened Species (2025). The species photographs were taken by David V. Raju, Wikimedia Commons ( *A. ellioti* ), Amitava Majumder ( *T. belangeri* ), and G. Gokulakrishnan ( *T. nicobarica* ).

Treeshrews are small, slender mammals, some being arboreal, some semi‐arboreal, while others are entirely terrestrial or scansorial (Martin et al. 2011; Hawkins 2018). They superficially resemble squirrels but can be differentiated by their elongated snout, hairless and moist nasal pad, and the lack of whiskers on their cheeks with their insectivorous and frugivorous feeding habits (Hawkins 2018). Their zoogeographical distribution is predominantly confined to the oriental region, being endemic to South and Southeast Asia (Lyon 1913). This region encompasses three major biodiversity hotspots: Sundaland, Indo‐Burma, and the Philippines (Myers et al. 2000; Sargis et al. 2013). Although treeshrews resemble squirrels, they were historically classified as primates (Carlsson 1922; Napier and Napier 1967; Sargis et al. 2013). However, they were eventually recognized as separate and assigned to their own order, Scandentia (Butler 1972; Helgen 2005). The order Scandentia comprises 23 treeshrew species under two families (Tupaiidae and Ptilocercidae); the family Tupaiidae contains three genera: *Tupaia*, *Dendrogale*, and the monotypic *Anathana* (Hawkins 2018). South Asian treeshrews include the monotypic Madras Treeshrew *Anathana ellioti* (Waterhouse 1850), the Northern Treeshrew *Tupaia belangeri* (Wagner 1841), and the Nicobar Treeshrew *Tupaia nicobarica* (Zelebor 1869). All three of these species occur in distinct and non‐overlapping geographical areas in India and Southeast Asia, where *Anathana ellioti* is restricted to peninsular India, *Tupaia belangeri* occurs throughout most North‐eastern India and Southeast Asia, and *Tupaia nicobarica* is confined to the Great and Little Nicobar Islands in the Andaman & Nicobar Islands, India (Burgin et al. 2018; Hawkins 2018). (Figure 1).

**FIGURE 2:**
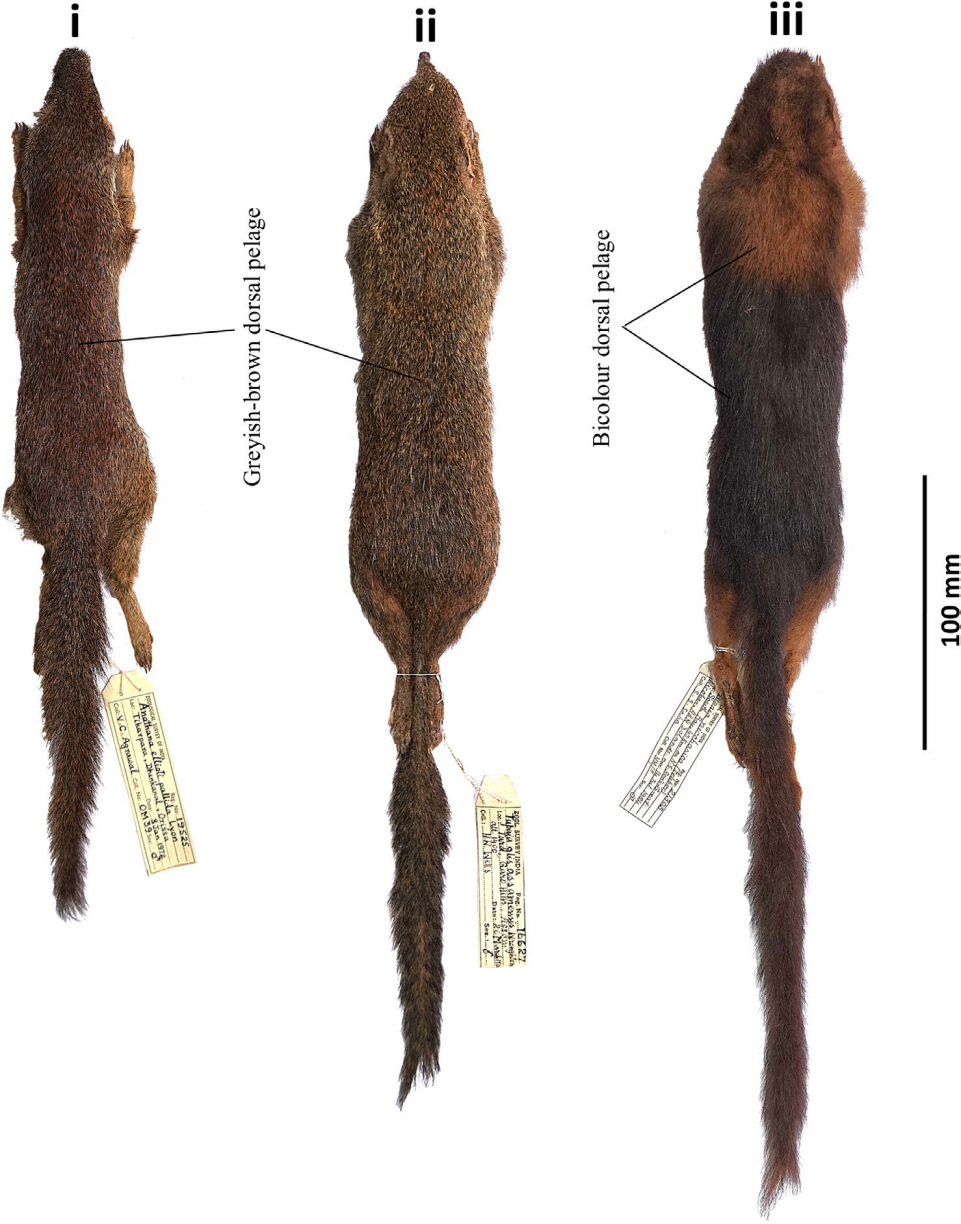
Dorsal views of study skins of South Asian treeshrews: (i) *Anathana ellioti* (ZSI 19525), (ii) *Tupaia belangeri* (ZSI 16627), and (iii) *Tupaia nicobarica* (ZSI 21305), archived at the Mammal & Osteology Section, Zoological Survey of India, Kolkata, India.

The taxonomy of treeshrews has been historically complicated by ambiguous morphological species boundaries (Helgen 2005; Sargis et al. 2013), and the treeshrews of South Asia are no exception. Identifying individual animals at the species level is crucial for managing conservation efforts and developing policy (Hawkins 2018). In India, the species identification of these treeshrews has primarily been determined based on the geographical origin of the samples. Among three South Asian treeshrew species, the island species *T. nicobarica* possesses the unique color of dorsal body pelage (bicolour reddish brown and blackish brown), while the mainland species *A. ellioti* and *T. belangeri* exhibit similar grayish‐brown dorsal pelage (Figure 2). Although *A. ellioti* and *T. belangeri* are recognized as two distinct species, they often lack morphological distinction. Hence, the body size and cranial measurements are used to differentiate these two species, besides their distinct geographical distribution.

The craniometric variation of *A. ellioti* (Juman et al. 2024) and *T. belangeri* (Juman et al. 2022a) has been examined in greater detail and compared with the six other treeshrew species, namely, *Dendrogale melanura* (Juman et al. 2024), *T. glis* (Sargis et al. 2013, 2014, 2017, 2018), *T. minor* (Juman et al. 2022b), *T. palawanensis* (Sargis et al. 2014), *T. tana* (Juman et al. 2021a), and *Ptilocercus lowii* (Juman et al. 2021b). However, the craniometric variation of *T. nicobarica* remained unexplored up to date. In the present study, we investigated morphological variations, including body size and craniomandibular measurements, in all three South Asian treeshrew species using distance‐based multivariate analyses and compared these data with the existing published datasets of *A. ellioti, T. belangeri, T. glis*, *T. minor* , *T. palawanensis* , *T. tana* , and *Ptilocercus lowii* (Sargis et al. 2013, 2014, 2017, 2018; Juman et al. 2021a, b, 2022a, b, 2024). Additionally, we also assessed the intraspecific morphometric variations among the previously recognized allopatric subspecies of *T. belangeri* (*T. b. assamensis*, *T. b. belangeri*, *T. b. brunetta*, *T. b. clarissa*, *T. b. lepcha*, and *T. b. siccata*) (Juman et al. 2022a) and *A. ellioti* (*A. e. ellioti* and *A.e. pallida*) (Juman et al. 2024).

## 2 Materials and Methods

Cranial measurements (hereafter referred to as CM) were obtained from a total of only 43 specimens (Appendix 1), body measurements (hereafter referred to as BM) from 61 specimens (Appendix 2), housed at the National Zoological Collections of the Mammal & Osteology Section, Zoological Survey of India (ZSI), representing *Anathana ellioti* (CM‐8; BM‐15), *Tupaia belangeri* (CM‐21; BM‐32), and *Tupaia nicobarica* (CM‐14; BM‐14), collected from the Andaman & Nicobar Islands and the mainland of India and few locations of Myanmar (Appendixs 1, 2). Only adult specimens were considered in this study, as indicated by the presence of a complete set of permanent teeth (Shigehara 1980; Woodman et al. 2020) and the fusion of the basioccipital suture (Martin et al. 2011). Specimens were identified by the museum catalogs and morphometric keys (Martin et al. 2011; Lekagul and McNeely 1977; Juman et al. 2022a; Juman et al. 2024). The first author recorded 22 craniomandibular measurements using Mitutoyo digital calipers that read to 0.01 mm (Sargis et al. 2013, 2014, 2017, 2018; Juman et al. 2021a, b, 2022a, b, 2024), and four body measurements using a 30 cm ruler (Martin et al. 2011). The body weights of only four specimens of *T. nicobarica* were recorded from the skin tags among all the specimens studied. All experimental procedures and techniques were used in compliance with the applicable rules, regulations, and authorization granted by the Zoological Survey of India.

Our samples also included previously recognized taxa now synonymized with *A. ellioti* : *A. e. ellioti*: (*n* = CM‐3; BM‐6) and *A. e*. *pallida* (*n* = CM‐5; BM‐9); *T. belangeri* : *T. b. assamensis* (*n* = CM‐9; BM‐13), *T. b. belangeri* (*n* = CM‐3; BM‐3), *T. b. brunetta* (*n* = CM‐2; BM‐5), *T. b. clarissa* (*n* = CM‐2; BM‐3), *T. b. lepcha* (*n* = CM‐5; BM‐5), and *T. b. siccata* (*n* = BM‐3) (see Supporting Information1; Appendixs 1, 2). The specimens studied were collected from 1851 to 1991. Global data for the same variables (excluding LB and LTPL due to unavailability) were gathered from previously published datasets on seven treeshrew taxa: *A. ellioti* (*n* = 21; Juman et al. 2024), *T. belangeri* (*n* = 351; Juman et al. 2022a), *T. glis* (*n* = 269; Sargis et al. 2013, 2014, 2017, 2018), *T. minor* (*n* = 37; Juman et al. 2022b), *T. palawanensis* (*n* = 31; Sargis et al. 2014), *T. tana* (*n* = 115; Juman et al. 2021a), and *Ptilocercus lowii* (*n* = 36; Juman et al. 2021b). Craniomandibular data from *Dendrogale melanura* could not be included in the analysis since only eight variables were available, as reported by Juman et al. (2024).

Descriptions of measurements # 1 to 20 (along with their abbreviations) as outlined by Sargis et al. (2013, 2014, 2017, 2018) and Juman et al. (2021a, b, 2022a, b, 2024): (1) condylo‐premaxillary length (CPL): greatest distance between the rostral surface of the premaxilla and the caudal surface of the occipital condyle; (2) condylo‐incisive length (CIL): greatest distance between the anterior‐most surface of I1 and the caudal surface of the occipital condyle; (3) upper toothrow length (UTL): greatest distance between the anterior‐most surface of I1 and the posterior‐most surface of M3; (4) maxillary toothrow length (MTL): greatest distance between the anterior‐most surface of C1 and the posterior‐most surface of M3; (5) palato‐premaxillary length (PPL): greatest distance between the rostral surface of the premaxilla and the caudal surface of the palatine; (6) mastoid breadth (MB): greatest distance between the lateral apices of the mastoid portion of the petrosal; (7) lacrimal breadth (LB): greatest distance between the lateral apices of the lacrimal tubercles; (8) least interorbital breadth (LIB): least distance between the orbits; (9) zygomatic breadth (ZB): greatest distance between the lateral surfaces of the zygomatic arch; (10) braincase breadth (BB): greatest breadth of the braincase; (11) lambdoid‐premaxillary length (LPL): greatest distance between the rostral surface of the premaxilla and the caudal surface of the lambdoid crest; (12) condylo‐nasal length (CNL): greatest distance between the rostral surface of the nasal and the caudal surface of the occipital condyle; (13) postorbital bar‐premaxillary length (PBPL): greatest distance between the rostral surface of the premaxilla and the caudal surface of the postorbital bar; (14) lacrimal tubercle‐premaxillary length (LTPL): greatest distance between the rostral surface of the premaxilla and the caudal surface of the lacrimal tubercle; (15) lambdoid crest height (LCH): greatest distance from apex (or apices if bilobate) of lambdoid crest to both ventral apices of occipital condyles (i.e., along midline); (16) mandibular height (MH): greatest distance between the coronoid and angular processes of the mandible; (17) mandibular condyle height (MCH): greatest distance between the mandibular condyle and the angular process of the mandible; (18) mandibular condyle width (MCW): greatest distance between the medial and lateral surfaces of the mandibular condyle; (19) mandibular condylo‐incisive length (MCIL): greatest distance between the anterior‐most surface of i1 and the caudal surface of the mandibular condyle; (20) lower toothrow length (LTL): greatest distance between the anterior‐most surface of i1 and the posterior‐most surface of m3; (21) orbit to interorbital region breadth (IOB): greatest distance between the orbit and the interorbital region; (22) mandibular coronoid breadth (MCB): greatest distance from the right to the left coronoid process of the mandible (# 21 and 22 are own records of the authors).

The measurements mentioned above were solely utilized, and identical descriptions were applied to existing published datasets to perform morphometric comparisons that were reported by Sargis et al. (2013, 2014, 2017, 2018) and Juman et al. (2021a, b, 2022a, b, 2024). As treeshrews typically do not exhibit sexual size dimorphism (Emmons 2000; Woodman et al. 2020; Juman et al. 2024), we therefore merged males and females in all analyses. Twenty craniomandibular measurements (variables) were classified into seven craniomandibular measurements based on broad structural regions of the skull for the multivariate analysis: (1) cranium length (CPL, CIL, LPL, and CNL); (2) skull breadth (ZB and BB); (3) mandibular height (MH and MCH); (4) mandibular length (MCIL and LTL); (5) maxilla length (UTL, MTL, PPL, and PBPL), with the remaining characters analyzed individually as described. The four body measurements include (1) head and body length (HBL): greatest distance between the tip of the snout to the base of the tail; (2) tail length (TL): greatest distance between the tip of the tail to its base adjacent to the body; (3) ear length (EL): greatest distance between the lower border of the external auditory meatus to the tip of the pinna; and (4) hind foot length (HFL): greatest distance between the extremity of the heel behind the os calcis to the extremity of the longest digit, not including the claws.

### 2.1 Statistical Analyses

Univariate analyses, including Levene's test for assessing the homogeneity of variance and Tukey's test, were performed using PAST version 2.15 (Hammer et al. 2001). Multivariate analyses, including principal component analysis (PCA), Bray–Curtis cluster analysis, and Mondrian plot based on the recorded craniomandibular traits, were carried out using PRIMER version 7.0.5 (Clarke and Gorley 2015). PCA was utilized to determine the principal morphometric variables that differentiate one species from another. A Mondrian plot was created by clustering samples and variables. The Bray–Curtis cluster analysis was studied to assess the similarities between the three species of treeshrews.

## 3 Results

### 3.1 Morphological Differentiation Among Three South Asian Treeshrews

**FIGURE 3:**
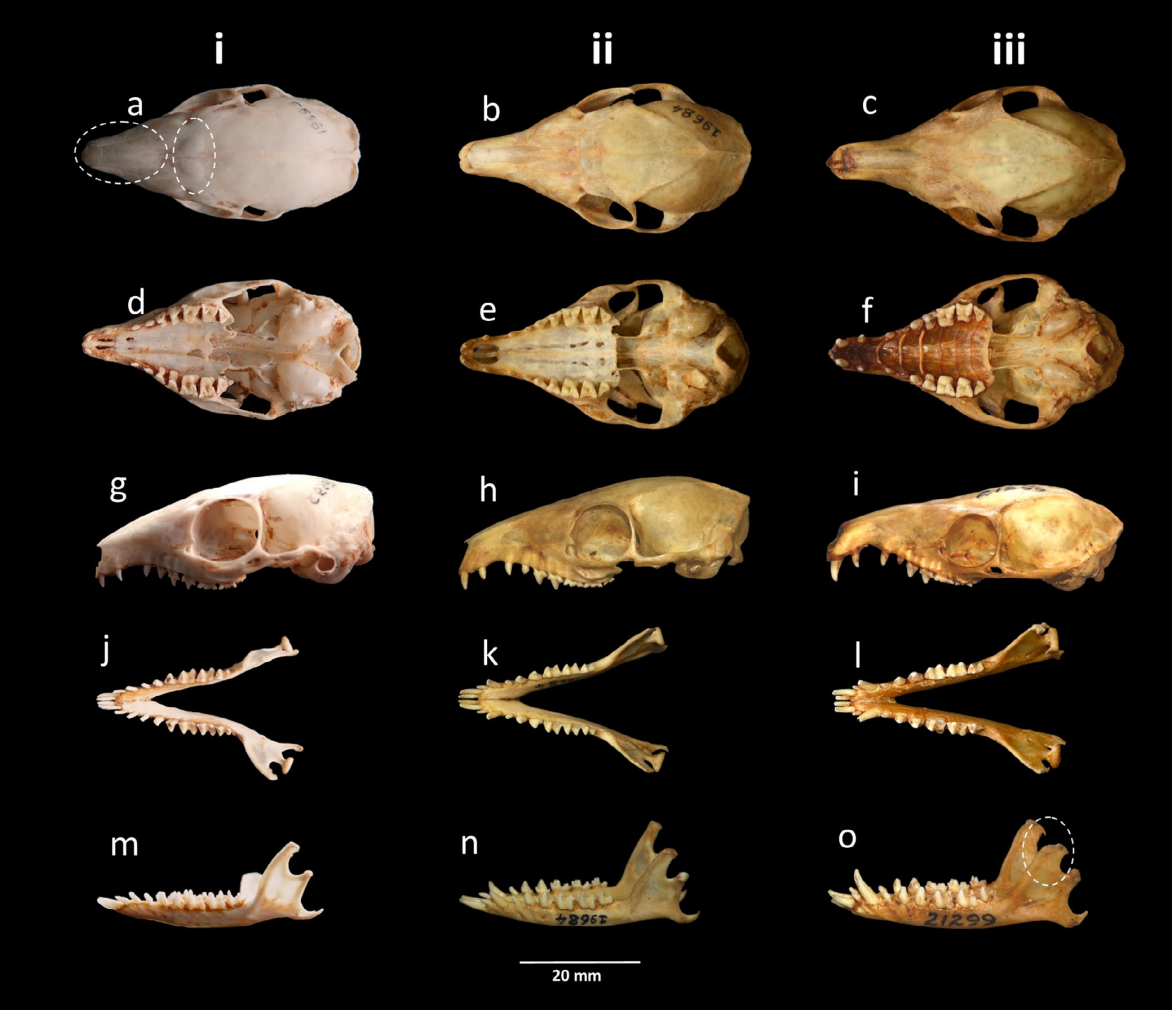
Cranium and mandible of South Asian treeshrews (i) *Anathana ellioti* (ZSI 19691), (ii) *Tupaia belangeri* (ZSI 19684), and (iii) *Tupaia nicobarica* (ZSI 21299), archived at the Mammal & Osteology Section, Zoological Survey of India, Kolkata, India. From top to bottom, a, b, c) dorsal, (d–f) ventral, (g–i) lateral views of the cranium, and (j–l) occlusal, and (m–o) lateral views of the mandible.

**FIGURE 4:**
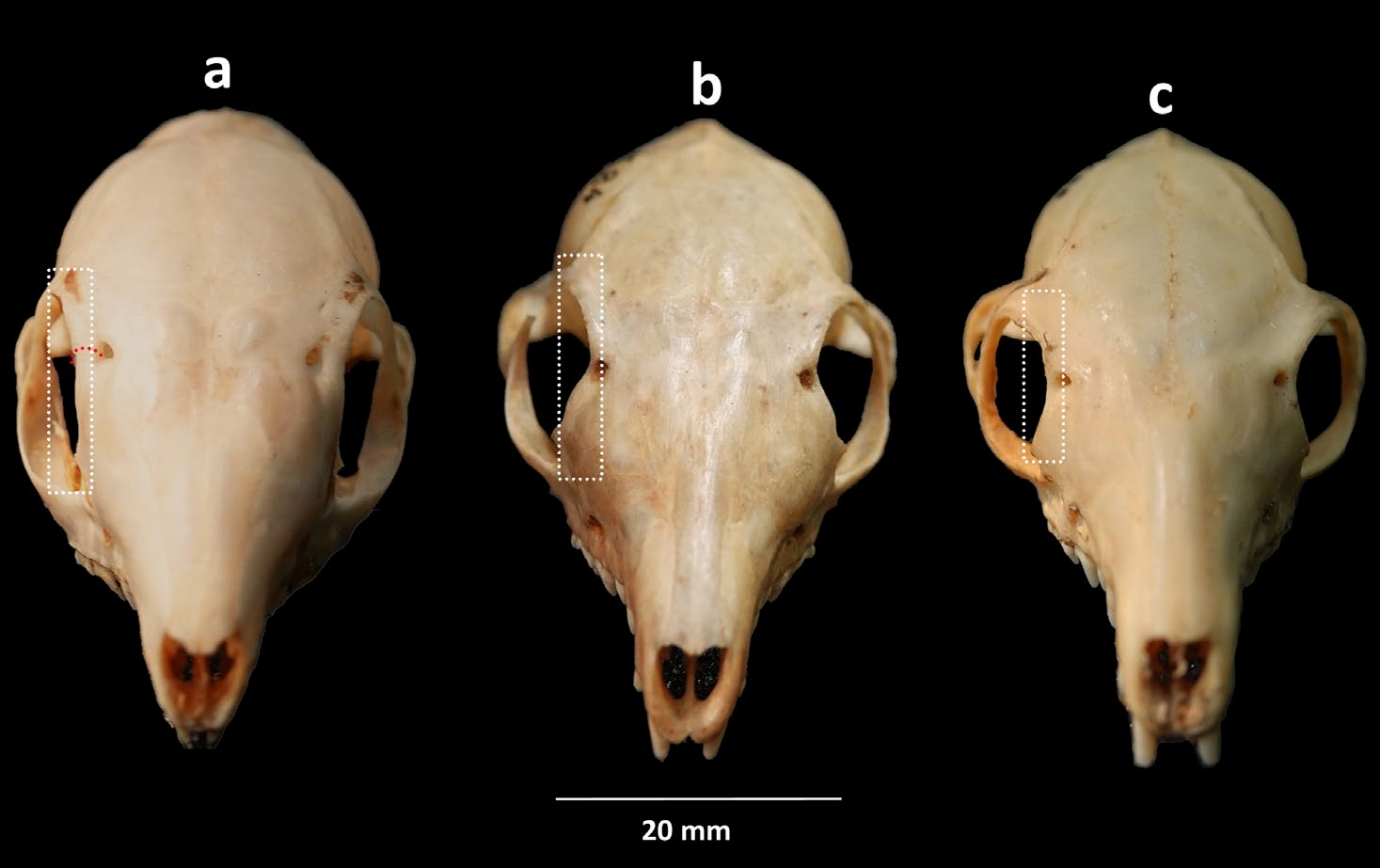
Anterior view of the cranium of South Asian treeshrews (a) *Anathana ellioti* (ZSI 19691), (b) *Tupaia belangeri* (ZSI 19684), and (c) *Tupaia nicobarica* (ZSI 21299), archived at the Mammal & Osteology Section, Zoological Survey of India, Kolkata, India. The white rectangular box (a, b, c) indicating the structure of orbit towards to the interorbital region, and a red curve (a) indicates wider distance between the interorbital region and the orbit in *A. ellioti* .

The craniomandibular characters clearly distinguished the three species of treeshrews (Figure 3), and we found the skull length of *Tupaia nicobarica* (LPL: 53.7 ± 0.79, 52.4–54.9 mm; MCIL: 39.7 ± 0.46, 39.1–40.3 mm) was larger than *T. belangeri* (LPL: 48.3 ± 1.3, 45.5–50.2 mm; MCIL: 30.8 ± 0.3, 35.4 ± 1.2, 32.8–37.2 mm) followed by *Anathana ellioti* (LPL: 42.5 ± 0.9, 41.2–43.6 mm; MCIL: 30.2–31.2 mm) (see Table 1; Supporting Information1). All three species possess a complete set of teeth (dental formula: incisor 2/3, canine 1/1, premolar 3/3 and molar 3/3×2 = 38; Figure 3d–f,j–l). The rostrum was relatively shorter and the maxilla‐frontal bone was slightly protruded in *A. ellioti* (Figure 3a) compared to *T. belangeri* and *T. nicobarica*. Further, the distance between orbits and interorbital regions was slightly wider in *A. ellioti* (IOB: 1.46 ± 0.09; 1.38–1.67 mm; Figure 4a) compared to *T. belangeri* (IOB: 0.69 ± 0.09; 0.56–0.88 mm; Figure 4b) and *T. nicobarica* (IOB: 0.90 ± 0.09; 0.80–1.09 mm; Figure 4c), and the structure of orbits was straight towards the interorbital regions in *A. ellioti* (Figure 4a), while it was notably curved in *T. belangeri* (Figure 4b) and slightly curved in *T. nicobarica* (Figure 4c). In addition, coronoid breadth in *T. nicobarica* was considerably greater (MCB: 3.47 ± 0.26; range: 3.02–3.85 mm; Table 1; Figure 3o) when compared to *T. belangeri* (IOB: 2.25 ± 0.19; range: 2.01–2.76 mm) and *A. ellioti* (MCB: 1.68 ± 0.15; range: 1.44–1.90 mm) (see Table 1; Supporting Information1).

**TABLE 1:** Summary statistics for the 22 craniomandibular measurements (mm), including mean ± SD, range of measurements, and sample sizes (n). Abbreviations for measurements are defined in the Materials and Methods section.

Species	(1) CPL	(2) CIL	(3) UTL	(4) MTL	(5) PPL	(6) MB	(7) LB	(8) LIB	(9) ZB	(10) BB	(11) LPL
***A. ellioti***	41.02 ± 0.78 39.75–42.13 (8)	40.42 ± 0.65 39.15–41.14 (8)	21.63 ± 0.42 20.80–22.09 (8)	14.81 ± 0.57 13.55–15.28 (8)	23.17 ± 0.48 22.36–23.75 (8)	16.25 ± 0.45 15.60–16.76 (8)	13.78 ± 0.43 13.20–14.42 (8)	13.33 ± 0.35 12.88–13.81 (8)	21.67 ± 0.56 20.870–22.40 (8)	17.34 ± 0.64 16.04–18.01 (8)	42.56 ± 0.95 41.24–43.65 (8)
***T. belangeri***	45.63 ± 1.41 42.98–47.39 (21)	45.13 ± 1.43 42.58–47.10 (21)	25.01 ± 0.94 23.02–26.79 (21)	17.30 ± 0.57 16.22–18.23 (21)	26.69 ± 1.03 24.89–28.56 (21)	17.49 ± 0.49 16.33–18.30 (21)	17.18 ± 0.87 15.49–18.43 (21)	13.59 ± 0.61 12.24–14.87 (21)	24.77 ± 1.07 22.23–26.53 (21)	19.11 ± 0.52 17.91–20.07 (21)	48.35 ± 1.33 45.51–50.29 (21)
***T. nicobarica***	49.82 ± 0.68 48.78–50.85 (14)	49.33 ± 0.62 48.41–50.26 (14)	28.07 ± 0.62 27.30–29.45 (14)	19.12 ± 0.36 18.42–19.67 (14)	29.66 ± 0.63 28.46–30.62 (14)	18.26 ± 0.4 17.61–18.83 (14)	18.34 ± 0.79 16.41–19.42 (14)	16.48 ± 0.63 15.51–17.44 (14)	28.25 ± 0.96 25.54–29.41 (14)	20.15 ± 0.46 19.19–20.82 (14)	53.71 ± 0.79 52.40–54.97 (14)

Species	(12) CNL	(13) PBPL	(14) LTPL	(15) LCH	(16) MH	(17) MCH	(18) MCW	(19) MCIL	(20) LTL	(21) IOB	(22) MCB
***A. ellioti***	40.68 ± 0.73 39.62–41.63 (8)	26.71 ± 0.44 26.11–27.57 (8)	16.92 ± 0.34 6.61–17.40 (8)	11.63 ± 0.28 11.16–12.05 (8)	11.75 ± 0.49 1.23–12.57 (8)	6.48 ± 0.66 5.51–7.75 (8)	2.54 ± 0.18 2.37–2.92 (8)	30.84 ± 0.35 30.24–31.28 (8)	20.64 ± 0.46 19.62–21.19 (8)	1.46 ± 0.09 1.38–1.67 (8)	1.68 ± 0.15 1.44–1.90 (8)
***T. belangeri***	44.39 ± 1.31 41.99–46.20 (21)	31.58 ± 1.21 29.20–33.92 (21)	22.87 ± 1.90 19.10–26.96 (21)	11.55 ± 0.37 10.90–12.36 (21)	13.19 ± 0.86 11.88–14.79 (21)	7.91 ± 0.84 6.15–9.01 (21)	3.20 ± 0.29 2.60–3.75 (21)	35.48 ± 1.27 32.85–37.25 (21)	23.57 ± 0.98 21.76–25.47 (21)	0.69 ± 0.09 0.56–0.88 (21)	2.25 ± 0.19 2.01–2.76 (21)
***T. nicobarica***	48.06 ± 0.86 46.49–49.30 (14)	33.35 ± 0.87 31.94–35.26 (14)	25.50 ± 0.68 24.57–26.59 (14)	12.31 ± 0.36 11.80–12.93 (14)	15.80 ± 0.4 15.05–16.50 (14)	10.03 ± 0.57 8.63–10.69 (14)	3.97 ± 0.19 3.61–4.24 (14)	39.72 ± 0.464 39.10–40.33 (14)	26.10 ± 0.57 25.11–27.16 (14)	0.90 ± 0.09 0.80–1.09 (14)	3.47 ± 0.26 3.02–3.85 (14)

The mean body‐linear measurements indicated that the head and body, as well as tail lengths of *T. nicobarica* , were relatively larger (HBL: 193.3 ± 12.99, 180–224 mm; TL: 226.1 ± 10.02, 210.5–240 mm) than those of *T. belangeri* (HBL: 171.3 ± 11.1, 148–189 mm; TL: 161 ± 10.59, 140–181 mm), followed by *A. ellioti* (HBL: 170.9 ± 11.7, 152–192 mm; TL: 181.8 ± 9.24, 165–195 mm). In *T. nicobarica* , the tail length exceeded the head and body lengths, while in *T. belangeri* and *A. ellioti* that they were nearly equal to their head and body lengths. Nevertheless, the ear length and hind foot length displayed considerable overlap among the three species investigated (Table 2).

**TABLE 2:** Summary statistics for the five body measurements (mm), including mean ± SD, range of measurements, and sample sizes (n). Abbreviations for measurements are defined in the Materials and Methods section.

Species	HBL	TL	EL	HFL	Body weight (g)
***A. ellioti***	170.9 ± 11.7 152–192 (15)	181.8 ± 9.24 165–195 (15)	16.08 ± 0.99 14–17.5 (15)	39.82 ± 1.46 37.29–43 (15)	Not available
***T. belangeri***	171.3 ± 11.1 148–189 (32)	161 ± 10.59 140–181 (32)	15.91 ± 1.63 11.7–22 (32)	40.28 ± 3.04 30–45 (32)	Not available
***T. nicobarica***	193.3 ± 12.99 180–224 (14)	226.1 ± 10.02 210.5–240 (14)	17.38 ± 1.56 12.6–19 (14)	45.01 ± 1.53 42.8–47 (14)	173.25 ± 17.95 140–190 (4)

### 3.2 Univariate and Multivariate Analyses

**FIGURE 5:**
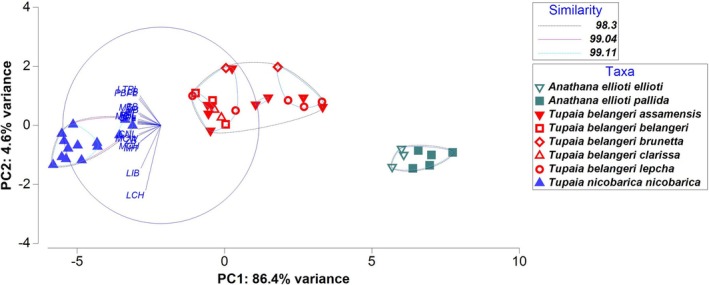
Principal component analysis of South Asian treeshrews based on cranium and mandible variables (refer to the Materials and Methods section for a detailed explanation of variable codes).

**FIGURE 6:**
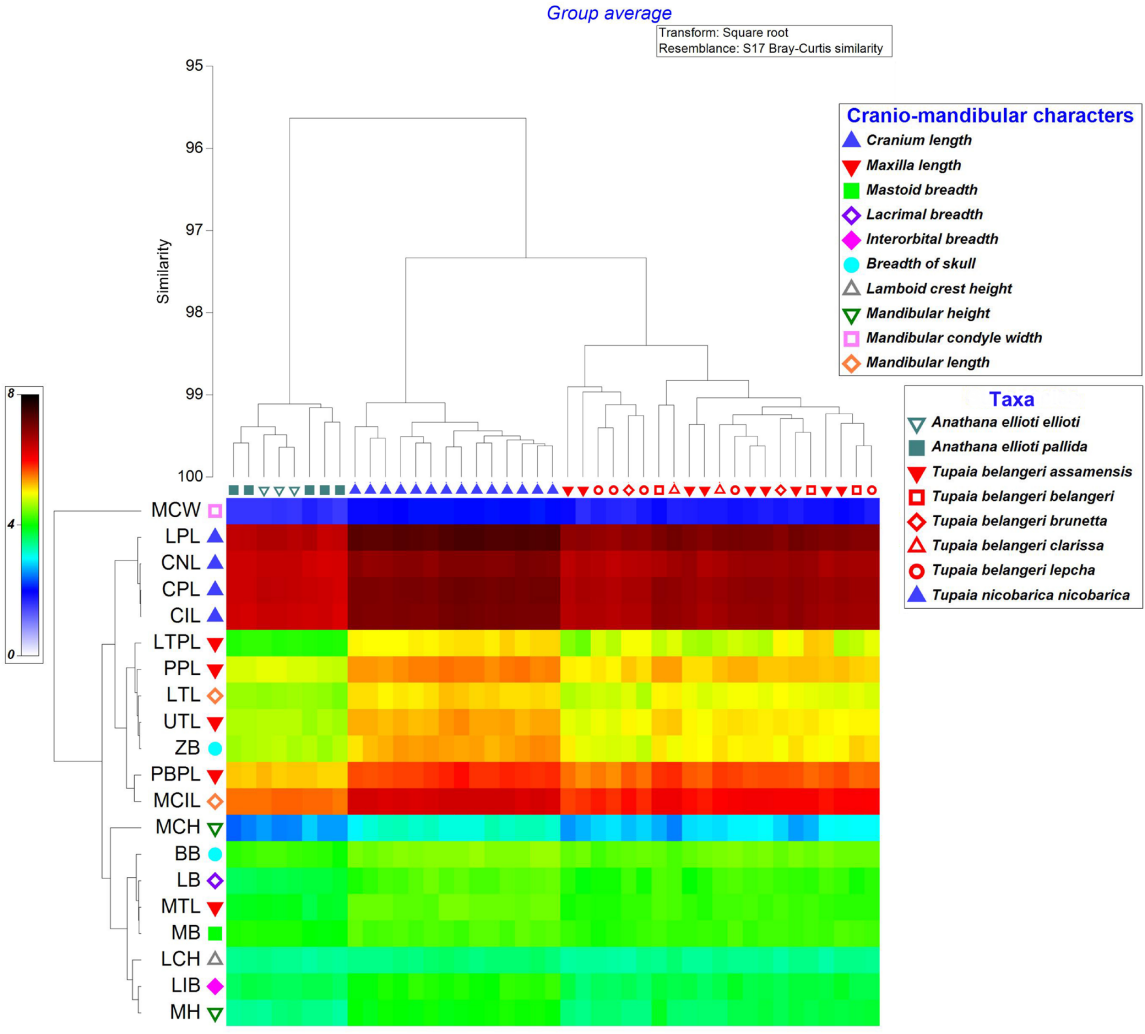
A Mondrian plot of South Asian treeshrews based on cranium and mandible variables (refer to the Materials and Methods section for a detailed explanation of variable codes).

PCA conducted on the craniometric data (Figure 5; Table 3) indicated that there was a strong variability (PC1: 86.4% variance) among the three species of South Asian treeshrews, with total cranium length being the most influenced trait observed. Based on these characters, the Tukey's pairwise comparisons regarding the differences among the species ( *A. ellioti* vs. *T. belangeri* , *T. belangeri* vs. *T. nicobarica* , and *A. ellioti* vs. *T. nicobarica* ) were determined to be statistically significant (*p* < 0.001). There was also a less variability observed (PC2: 4.6% variance), which is influenced by the length of the Lacrimal tubercle to the premaxilla (LTPL). Similarly, Tukey's test related to the variation among species based on LTPL showed significant results (*p* < 0.001). With the exception of LPL, the principal components observed within the species also exhibited significant differences (*p* < 0.05) according to Levene's test for homogeneity of variance. Furthermore, Tukey's test showed a significant difference in variation among the species regarding the orbit to interorbital region breadth (IOB) and the mandibular coronoid breadth (MCB) (*p* < 0.001). Additionally, it was observed that there were no significant differences (*p* > 0.05) in Levene's test for homogeneity of variance concerning IOB and MCB traits within the samples of each species. The Bray–Curtis cluster analysis (Figure 6) revealed three distinct clusters corresponding to the three species. The first major cluster comprised *A. ellioti* , merging *A. e. ellioti* and *A. e. pallida* with 99.11% similarity. The second cluster included five subspecies of *T. belangeri* (*T. b. assamensis*, *T. b. belangeri*, *T. b. brunetta*, *T. b. clarissa*, and *T. b. lepcha*), showing 99.04% similarity. The third cluster was formed by *T. nicobarica* , exhibiting 98.3% similarity. The Mondrian plot further supported the identification of key cranio‐mandibular variables responsible for distinguishing Indian treeshrew species. Among these, variables related to cranium length played a crucial role in defining these clusters and differentiating between species.

**TABLE 3:** Eigenvalues and percent of total variance from PCA of South Asian treeshrew species.

Eigenvalues			
PC	Eigenvalues	%Variation	Cumulative % Variation
1	17.3	86.4	86.4
2	0.921	4.6	91.0
3	0.493	2.5	93.5
4	0.303	1.5	95.0
5	0.232	1.2	96.1
Eigenvectors (Coefficients in the linear combinations of variables making up PC's)			

Variable	PC1	PC2
LPL	−0.238	0.014
CPL	−0.238	0.015
CIL	−0.238	0.027
UTL	−0.236	0.048
MTL	−0.233	0.120
PPL	−0.237	0.041
MB	−0.213	0.097
LB	−0.217	0.sss
LIB	−0.204	**−0.437**
ZB	−0.230	−0.104
BB	−0.219	0.128
CNL	−0.234	−0.037
PBPL	−0.224	0.267
LTPL	−0.215	**0.304**
LCH	−0.156	−0.661
MH	−0.225	−0.182
MCH	−0.207	−0.161
MCW	−0.223	−0.088
MCIL	−0.237	0.032
LTL	−0.233	0.063

*Note:* Emphasized values represent the weighted components of the eigenvectors corresponding to PC1 and PC2.

**FIGURE 7:**
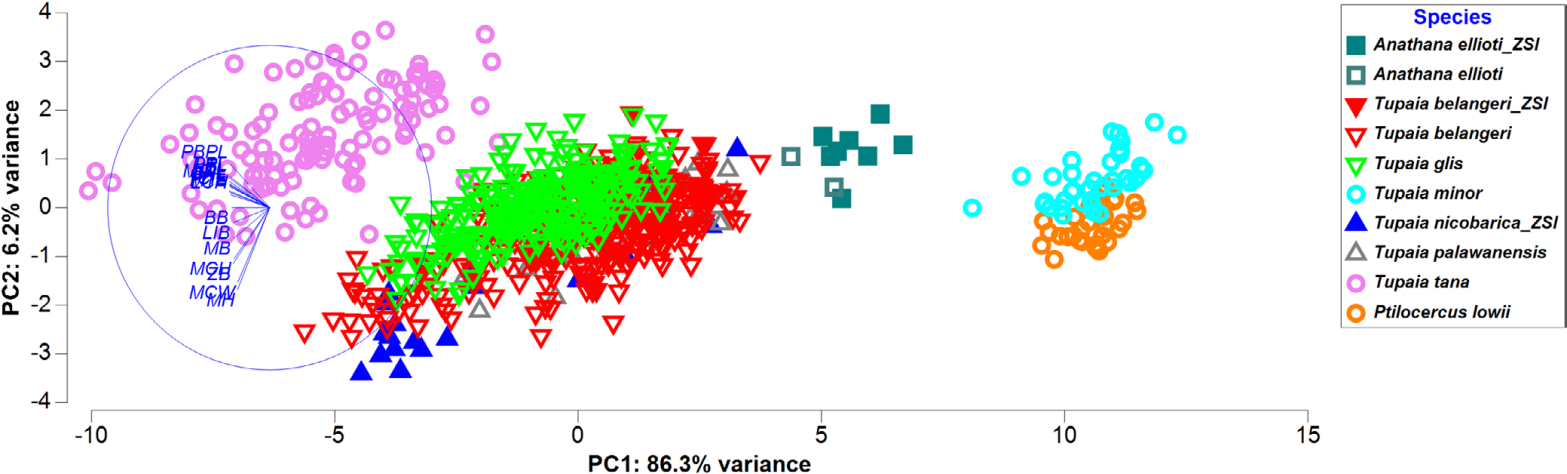
Principal component analysis of global treeshrew species including South Asian treeshrews based on cranium and mandible variables (refer to the Materials and Methods section for a detailed explanation of variable codes).

The craniometric measurements of the three South Asian treeshrew species were analyzed alongside five other treeshrew taxa from Southeast Asia using previously published datasets (Sargis et al. 2013, 2014, 2017, 2018; Juman et al. 2021a, b, 2022a, b, 2024). The PCA analysis (Figure 7; Table 4) indicated variability (PC2: 6.2% variance) among the species, with the contributing characteristics being linked to differences in zygomatic breadth (ZB), mandibular height (MH), mandibular condyle height (MCH), and mandibular condyle width (MCW).

**TABLE 4:** Eigenvalues and percent of total variance from PCA of South Asian and other five treeshrew species.

Eigenvalues			
PC	Eigenvalues	%Variation	Cumulative % Variation
1	15.5	86.3	86.3
2	1.11	6.2	92.5
3	0.354	2.0	94.5
4	0.267	1.5	95.9
5	0.221	1.2	97.2
Eigenvectors (Coefficients in the linear combinations of variables making up PC's)			

Variable	PC1	PC2
CPL	−0.249	0.136
CIL	−0.250	0.104
UTL	−0.248	0.160
MTL	−0.247	0.094
PPL	−0.245	0.192
MB	−0.222	−0.190
LIB	−0.225	−0.092
ZB	−0.225	**−0.344**
BB	−0.234	0.000
LPL	−0.249	0.143
CNL	−0.246	0.130
PBPL	−0.240	0.265
LCH	−0.240	0.079
MH	−0.199	**−0.508**
MCH	−0.220	**−0.315**
MCW	−0.197	**−0.460**
MCIL	−0.249	0.147
LTL	−0.247	0.180

*Note:* Emphasized values represent the weighted components of the eigenvectors corresponding to PC1 and PC2.

## 4 Discussion

Treeshrew species often appear very similar to one another, which can make it quite challenging to differentiate between them in their environments, making the clear definition of features that separate species quite useful (Sargis et al. 2014). Morphological traits have occasionally been used to delineate taxonomic boundaries among the treeshrews found in South Asia. While detailed morphological studies exist for some global treeshrew taxa (Sargis et al. 2013, 2014, 2017, 2018; Juman et al. 2021a, b, 2022a, b, 2024), comparative analyses of South Asian treeshrews have been limited due to the scarcity of museum specimens for *Anathana ellioti* (Juman et al. 2024) and the absence of samples for *Tupaia nicobarica* .

Our analyses of cranio‐mandibular traits and body measurements from museum specimens of mainland and island populations in India and Myanmar offered valuable insights, effectively differentiating the three species of South Asian treeshrews. Both cranial measurement and body size analyses (Figures 5, 6; Table 2) established that *T. nicobarica* is larger than *T. belangeri* and *A. ellioti* , which contradicts previous claims by Oommen (2013, pg. 62), who stated that “ *T. nicobarica* is the smallest among the three South Asian treeshrews, and perhaps the smallest among all tupaiids”. These earlier erroneous reports (Oommen 2013; Menon 2014) likely arose from either a scarcity of museum specimens for comparison or not being referred to the published literature. Roonwal and Mohnot (1977) listed *T. nicobarica* at 170 g, and Sargis (2002) listed several tupaiid species at lower weights. The average body weight of four *T. nicobarica* specimens (173.25 g; Table 2) also indicates that it is the third largest treeshrew species globally after *T. everetti* and *T. tana* (Sargis 2002). Moreover, the maximum coronoid breadth would make it significantly simpler to differentiate *T. nicobarica* from *A. ellioti* and *T. belangeri* at first glance (Table 1; Figure 3o). The larger body size of *T. nicobarica* may relate to the island effect on size (Foster's Island rule); this rule posits that larger mammals on islands tend to evolve smaller average body sizes compared to their mainland relatives, while smaller mammals typically evolve larger sizes (insular gigantism), although this “rule” does not seem to be consistently applicable to treeshrews (Juman et al. 2022a; Sargis et al. 2018). The applicability of the island rule, which posits that small mammals on islands often evolve larger sizes while large mammals become smaller, can be influenced by various ecological factors, including the presence or absence of predators (Lomolino 1985). Niche constraints also play a significant role in shaping body size evolution on islands. The limited availability of resources and reduced interspecific competition can lead to shifts in optimal body size (Benítez‐López et al. 2021). *T. tana* is the nearest known allopatric species to *T. nicobarica* , residing in Sumatra, which is approximately 180 km away from Nicobar Island, and both species inhabit the island ecosystem (Payne et al. 1985; Yasuma et al. 2003; Juman et al. 2021a). Although the Nicobar Islands are politically considered part of India, they are geographically part of Southeast Asia, and the Nicobar species ( *T. nicobarica* ) may have significant historical, geographical, and genetic connections with those found on Sumatra and the Malay Peninsula. Based on geographical distribution, two subspecies of *T. nicobarica* have been recognized, such as, *T. n. nicobarica* from the Great Nicobar Island and *T. n. surda* from the Little Nicobar Island (Helgen 2005). However, Kundu et al. (2022) reported that both subspecies maintained less genetic distance (0.7%) from each other based on *16S rRNA* genes (1667 bp) molecular studies.

Comparative relationships among the three species and with the other five species (Sargis et al. 2013, 2014, 2017, 2018; Juman et al. 2021a, b, 2022a, b, 2024; Figure 7) further emphasize size differences and also indicate how variation in individual measurements is distributed. *A. ellioti* continues to be smaller than most species (but larger than *T. minor* and *Ptilocercus lowii* ), which is consistent with the findings of Juman et al. (2024). *T. belangeri* and *T. nicobarica* show size overlap with *T. glis* and *T. palawanensis* , but they are different from smaller taxa like *T. minor* and *Ptilocercus lowii* . Our observations on *T. belangeri* corroborate those of Juman et al. (2022a).

As indicated by our multivariate analyses and supported by the works of Lyon (1913), Olson et al. (2005), Roberts et al. (2011), and Juman et al. (2024), we also recommend retaining the distinct genus categories of *Anathana* and *Tupaia* due to notable differences (Figures 3, 4; Tables 1, 2). Currently, no subspecies are recognized in *A. ellioti* or *T. belangeri* (Juman et al. 2022a, 2024; Oommen 2013). Our study results also indicate the same complete overlap among subspecies (Figures 5, 6).

In terms of external morphological characteristics and measurements, *A. ellioti* has a short and robust rostrum, while both *T. belangeri* and *T. nicobarica* possess long rostrums. This finding aligns with the original characterization by Lyon (1913) and a recent investigation by Juman et al. (2024). The two mainland treeshrew species, *A. ellioti* and *T. belangeri* , exhibit morphological similarities (Figure 2), especially in dorsal pelage; however, *A. ellioti* features a reticulated nose pad and a tail that is shorter than its head and body length, whereas *T. belangeri* has a somewhat elongated snout with a tail that matches the head and body length (Menon 2014). The body dimensions vary among collectors and can change depending on mortality conditions; these metrics were typically recorded by multiple collectors across various times and places (Sumner 1927; Blackwell et al. 2006; Stephens et al. 2015; Theriot et al. 2023). However, the mean of the external measurements (Table 2) will help to forecast the overall differences in linear length across the species studied.

The protrusions of the nuchal crest on the supraoccipital region, a key characteristic for distinguishing *A. ellioti* from its relatives, were observed in this study, as indicated by Juman et al. (2024). In addition, the comparatively broader premaxilla (Figure 3a) and slightly wider distance between the interorbital regions and the orbits, as well as the straight orientation of the orbit in *A. ellioti* (Figure 4a), can also be used as distinguishing characteristics for identification of this species, as noted in this study.

Based on their distribution across various regions in India, three subspecies were previously classified within the genus *Anathana*: *A. e. ellioti* in Southern India, *A. e. pallida* in central India, and *A. e. wroughtoni* in western India (Ellerman and Morrison‐Scott 1951). However, later studies did not recognize all three subspecies due to a limited number of samples, as noted by Helgen (2005) and Juman et al. (2024). Similarly, *Tupaia belangeri* had six previously recognized subspecies in South Asia: *T. b. assamensis* in Eastern India, *T. b. belangeri* in southern Myanmar, *T. b. clarissa* in southern Myanmar, *T. b. lepcha* in northern Bengal, *T. b. siccata* in the Shan state of Myanmar, and *T. b. versurae* in eastern India (Oommen 2013).

Our sampling includes two previously recognized subspecies of *A. ellioti* (excluding *wroughtoni*) and five subspecies of *T. belangeri* (excluding *versurae*). The results of our study indicate a complete overlap among them (Figures 5, 6), which further supports the recommendations made by Juman et al. (2022a; 2024) to maintain the synonymy of the subspecies of *A. ellioti* and *T. belangeri*. Future studies of the phylogeography among the South Asian treeshrews may provide relevant insights into the historical, geographical, and genetic relatedness of these species.

### 4.1 Conservation Implications

*T. nicobarica* is classified as ‘endangered’, and both *Anathana ellioti* and *T. belangeri* are classified as ‘least concern’ by the International Union for Conservation of Nature's Red List of Threatened Species (IUCN 2025). However, all three of these species are listed as Schedule I ( *T. belangeri* & *T. nicobarica* ) and Schedule II ( *A. ellioti* ) mammals under the Indian Wildlife (Protection) Amendment Act, 2022 (Kamalakannan et al. 2021; Sharma et al. 2024), and all three species are listed as Appendix 2 under the Convention on International Trade in Endangered Species of Wild Fauna and Flora. All treeshrews are listed under CITES because they used to be classified as Primates (e.g., Roonwal and Mohnot 1977). Our findings provide a robust framework for understanding variation within South Asian treeshrews, contributing to improved conservation and management efforts.

## Author Contributions

**Manokaran Kamalakannan:** conceptualization (lead), data curation (lead), formal analysis (lead), investigation (lead), methodology (lead), supervision (lead), validation (lead), visualization (lead), writing – original draft (lead), writing – review and editing (lead). **Mukesh Thakur:** methodology (supporting), resources (supporting), writing – review and editing (supporting). **Nithyanandam Marimuthu:** formal analysis (equal), software (lead), writing – review and editing (supporting). **Subhojit Pramanik:** data curation (supporting). **Dhriti Banerjee:** funding acquisition (lead), project administration (lead), writing – review and editing (supporting).

## Conflicts of Interest

The authors declare no conflicts of interest.

## Supporting information


Data S1.

## Data Availability

The datasets generated and analyzed during the current study are given in Supporting Information1.

## Appendix 1 List of Specimens Examined for Craniomandibular Analyses

**Table:**

Sl. No.	Institute	Reg. Number	Species labeled as	Species recognized as	General locality	Date of collection	Sex
1.	ZSI	7168	*Anathana ellioti ellioti*	*Anathana ellioti*	Tamil Nadu		
2.	ZSI	7170	*Anathana ellioti ellioti*	*Anathana ellioti*	Tamil Nadu		
3.	ZSI	7169	*Anathana ellioti ellioti*	*Anathana ellioti*	Tamil Nadu		
4.	ZSI	19,692	*Anathana ellioti pallida*	*Anathana ellioti*	Bihar	1.05.1880	M
5.	ZSI	19,693	*Anathana ellioti pallida*	*Anathana ellioti*	Bihar	1.05.1880	F
6.	ZSI	19,691	*Anathana ellioti pallida*	*Anathana ellioti*	Bihar	1.05.1880	M
7.	ZSI	19,526	*Anathana ellioti pallida*	*Anathana ellioti*	Odisha	28.5.1972	M
8.	ZSI	19,525	*Anathana ellioti pallida*	*Anathana ellioti*	Odisha	8.1.1972	M
9.	ZSI	18,864	*Tupaia belangeri assamensis*	*Tupaia belangeri*	Tripura	13.12.1972	M
10.	ZSI	19,684	*Tupaia belangeri assamensis*	*Tupaia belangeri*	Assam	28.01.1957	M
11.	ZSI	16,634	*Tupaia belangeri assamensis*	*Tupaia belangeri*	Meghalaya	10.04.1921	M
12.	ZSI	16,627	*Tupaia belangeri assamensis*	*Tupaia belangeri*	Meghalaya	26.3.1970	M
13.	ZSI	R/14	*Tupaia belangeri assamensis*	*Tupaia belangeri*	Manipur	14.11.1945	M
14.	ZSI	23,846	*Tupaia belangeri assamensis*	*Tupaia belangeri*	Mizoram	24.10.1991	F
15.	ZSI	11,136	*Tupaia belangeri assamensis*	*Tupaia belangeri*	Manipur	27.11.1945	M
16.	ZSI	11,135	*Tupaia belangeri assamensis*	*Tupaia belangeri*	Manipur	14.11.1945	M
17.	ZSI	17,989	*Tupaia belangeri assamensis*	*Tupaia belangeri*	Meghalaya	1857	
18.	ZSI	16,635	*Tupaia belangeri belangeri*	*Tupaia belangeri*	Myanmar	20.4.1916	M
19.	ZSI	16,636	*Tupaia belangeri belangeri*	*Tupaia belangeri*	Myanmar	19.6.1927	F
20.	ZSI	16,637	*Tupaia belangeri belangeri*	*Tupaia belangeri*	Myanmar	7.1.1928	F
21.	ZSI	16,631	*Tupaia belangeri brunetta*	*Tupaia belangeri*	Myanmar	29.10.1921	F
22.	ZSI	16,630	*Tupaia belangeri brunetta*	*Tupaia belangeri*	Myanmar	2.10.1921	M
23.	ZSI	16,638	*Tupaia belangeri clarissa*	*Tupaia belangeri*	Myanmar	19.07.1913	M
24.	ZSI	16,640	*Tupaia belangeri clarissa*	*Tupaia belangeri*	Myanmar	30.4.1914	M
25.	ZSI	16,625	*Tupaia belangeri lepcha*	*Tupaia belangeri*	West Bengal	1.4.1916	M
26.	ZSI	16,624	*Tupaia belangeri lepcha*	*Tupaia belangeri*	West Bengal	7.1.1916	F
27.	ZSI	16,626	*Tupaia belangeri lepcha*	*Tupaia belangeri*	West Bengal	21.6.1916	M
28.	ZSI	26,650	*Tupaia belangeri lepcha*	*Tupaia belangeri*	West Bengal		
29.	ZSI	16,623	*Tupaia belangeri lepcha*	*Tupaia belangeri*	West Bengal	20.3.1915	F
30.	ZSI	19,688	*Tupaia nicobarica nicobarica*	*Tupaia nicobarica*	Andaman & Nicobar Is.	15.12.1875	M
31.	ZSI	20,768	*Tupaia nicobarica nicobarica*	*Tupaia nicobarica*	Andaman & Nicobar Is.	30.03.1977	M
32.	ZSI	21,308	*Tupaia nicobarica nicobarica*	*Tupaia nicobarica*	Andaman & Nicobar Is.	30.07.1984	F
33.	ZSI	GNM 77/4	*Tupaia nicobarica nicobarica*	*Tupaia nicobarica*	Andaman & Nicobar Is.	29.07.1977	F
34.	ZSI	21,304	*Tupaia nicobarica nicobarica*	*Tupaia nicobarica*	Andaman & Nicobar Is.	4.07.1984	M
35.	ZSI	20,764	*Tupaia nicobarica nicobarica*	*Tupaia nicobarica*	Andaman & Nicobar Is.	24.04.1975	F
36.	ZSI	20,765	*Tupaia nicobarica nicobarica*	*Tupaia nicobarica*	Andaman & Nicobar Is.	29.04.1975	M
37.	ZSI	20,767	*Tupaia nicobarica nicobarica*	*Tupaia nicobarica*	Andaman & Nicobar Is.	29.03.1977	M
38.	ZSI	20,766	*Tupaia nicobarica nicobarica*	*Tupaia nicobarica*	Andaman & Nicobar Is.	30.04.1975	F
39.	ZSI	21,307	*Tupaia nicobarica nicobarica*	*Tupaia nicobarica*	Andaman & Nicobar Is.	30.07.1984	M
40.	ZSI	21,303	*Tupaia nicobarica nicobarica*	*Tupaia nicobarica*	Andaman & Nicobar Is.	30.06.1984	M
41.	ZSI	21,306	*Tupaia nicobarica nicobarica*	*Tupaia nicobarica*	Andaman & Nicobar Is.	17.07.1984	M
42.	ZSI	21,299	*Tupaia nicobarica nicobarica*	*Tupaia nicobarica*	Andaman & Nicobar Is.	14.07.1984	M
43.	ZSI	6535	*Tupaia nicobarica nicobarica*	*Tupaia nicobarica*	Andaman & Nicobar Is.		M

## Appendix 2 List of Specimens Examined for Body‐Morphometric Analyses

**Table:**

Sl. No.	Institute	Reg. No.	Species labeled as	Species recognized as	General locality	Date of collection	Sex
1.	ZSI	7168	*Anathana ellioti ellioti*	*Anathana ellioti*	Tamil Nadu		
2.	ZSI	7170	*Anathana ellioti ellioti*	*Anathana ellioti*	Tamil Nadu		
3.	ZSI	7169	*Anathana ellioti ellioti*	*Anathana ellioti*	Tamil Nadu		
4.	ZSI	19,692	*Anathana ellioti pallida*	*Anathana ellioti*	Bihar	1.05.1880	M
5.	ZSI	19,693	*Anathana ellioti pallida*	*Anathana ellioti*	Bihar	1.05.1880	F
6.	ZSI	19,691	*Anathana ellioti pallida*	*Anathana ellioti*	Bihar	1.05.1880	M
7.	ZSI	19,526	*Anathana ellioti pallida*	*Anathana ellioti*	Odisha	28.5.1972	M
8.	ZSI	19,525	*Anathana ellioti pallida*	*Anathana ellioti*	Odisha	8.1.1972	M
9.	ZSI	7231	*Anathana ellioti ellioti*	*Anathana ellioti*	Tamil Nadu		F
10.	ZSI	19,756	*Anathana ellioti ellioti*	*Anathana ellioti*	Tamil Nadu	1851	N
11.	ZSI	7171	*Anathana ellioti ellioti*	*Anathana ellioti*			
12.	ZSI	16,294	*Anathana ellioti pallida*	*Anathana ellioti*	Odisha	6.10.1921	F
13.	ZSI	202F	*Anathana ellioti pallida*	*Anathana ellioti*		1874	F
14.	ZSI	202E	*Anathana ellioti pallida*	*Anathana ellioti*		1874	M
15.	ZSI	202D	*Anathana ellioti pallida*	*Anathana ellioti*		1874	M
16.	ZSI	19,671	*Tupaia belangeri assammensis*	*Tupaia belangeri belangeri*	Arunachal Pradesh		
17.	ZSI	19,670	*Tupaia belangeri assammensis*	*Tupaia belangeri belangeri*	Assam		M
18.	ZSI	19,685	*Tupaia belangeri assammensis*	*Tupaia belangeri belangeri*			F
19.	ZSI	R2/14	*Tupaia belangeri assammensis*	*Tupaia belangeri belangeri*			M
20.	ZSI	18,865	*Tupaia belangeri assammensis*	*Tupaia belangeri belangeri*			M
21.	ZSI	19,686	*Tupaia belangeri assammensis*	*Tupaia belangeri belangeri*			M
22.	ZSI	19,685	*Tupaia belangeri assammensis*	*Tupaia belangeri belangeri*			F
23.	ZSI	18,864	*Tupaia belangeri assammensis*	*Tupaia belangeri belangeri*	Tripura	13.12.1972	M
24.	ZSI	19,684	*Tupaia belangeri assammensis*	*Tupaia belangeri belangeri*	Assam	28.01.1957	M
25.	ZSI	16,634	*Tupaia belangeri assammensis*	*Tupaia belangeri belangeri*	Meghalaya	10.04.1921	M
26.	ZSI	16,627	*Tupaia belangeri assammensis*	*Tupaia belangeri belangeri*	Meghalaya	26.3.1970	M
27.	ZSI	23,846	*Tupaia belangeri assammensis*	*Tupaia belangeri belangeri*	Mizoram	24.10.1991	F
28.	ZSI	11,136	*Tupaia belangeri assammensis*	*Tupaia belangeri belangeri*	Manipur	27.11.1945	M
29.	ZSI	16,635	*Tupaia belangeri belangeri*	*Tupaia belangeri belangeri*	Myanmar	20.4.1916	M
30.	ZSI	16,636	*Tupaia belangeri belangeri*	*Tupaia belangeri belangeri*	Myanmar	19.6.1927	F
31.	ZSI	16,637	*Tupaia belangeri belangeri*	*Tupaia belangeri belangeri*	Myanmar	7.1.1928	M
32.	ZSI	16,631	*Tupaia belangeri brunetta*	*Tupaia belangeri belangeri*	Myanmar	29.10.1921	F
33.	ZSI	16,630	*Tupaia belangeri brunetta*	*Tupaia belangeri belangeri*	Myanmar	2.10.1921	M
34.	ZSI	16,629	*Tupaia belangeri brunetta*	*Tupaia belangeri belangeri*			F
35.	ZSI	16,633	*Tupaia belangeri brunetta*	*Tupaia belangeri belangeri*			M
36.	ZSI	16,632	*Tupaia belangeri brunetta*	*Tupaia belangeri belangeri*			F
37.	ZSI	5247	*Tupaia belangeri clarissa*	*Tupaia belangeri belangeri*		21.4.1882	M
38.	ZSI	16,638	*Tupaia belangeri clarissa*	*Tupaia belangeri belangeri*	Myanmar	19.07.1913	M
39.	ZSI	16,640	*Tupaia belangeri clarissa*	*Tupaia belangeri belangeri*	Myanmar	30.4.1914	M
40.	ZSI	16,625	*Tupaia belangeri lepcha*	*Tupaia belangeri belangeri*	West Bengal	1.4.1916	M
41.	ZSI	16,624	*Tupaia belangeri lepcha*	*Tupaia belangeri belangeri*	West Bengal	7.1.1916	F
42.	ZSI	16,626	*Tupaia belangeri lepcha*	*Tupaia belangeri belangeri*	West Bengal	21.6.1916	M
43.	ZSI	26,650	*Tupaia belangeri lepcha*	*Tupaia belangeri belangeri*	West Bengal		
44.	ZSI	16,623	*Tupaia belangeri lepcha*	*Tupaia belangeri belangeri*	West Bengal	20.3.1915	F
45.	ZSI	16,622	*Tupaia belangeri siccata*	*Tupaia belangeri belangeri*			M
46.	ZSI	16,619	*Tupaia belangeri siccata*	*Tupaia belangeri belangeri*			M
47.	ZSI	16,618	*Tupaia belangeri siccata*	*Tupaia belangeri belangeri*			F
48.	ZSI	20,768	*Tupaia nicobarica nicobarica*	*Tupaia nicobarica nicobarica*	Andaman & Nicobar Island		M
49.	ZSI	21,308	*Tupaia nicobarica nicobarica*	*Tupaia nicobarica nicobarica*	Andaman & Nicobar Island	30.07.1984	F
50.	ZSI	GNM 77/4	*Tupaia nicobarica nicobarica*	*Tupaia nicobarica nicobarica*	Andaman & Nicobar Island	29.07.1977	F
51.	ZSI	21,304	*Tupaia nicobarica nicobarica*	*Tupaia nicobarica nicobarica*	Andaman & Nicobar Island	4.07.1984	M
52.	ZSI	20,764	*Tupaia nicobarica nicobarica*	*Tupaia nicobarica nicobarica*	Andaman & Nicobar Island	24.04.1975	F
53.	ZSI	20,765	*Tupaia nicobarica nicobarica*	*Tupaia nicobarica nicobarica*	Andaman & Nicobar Island	29.04.1975	M
54.	ZSI	20,767	*Tupaia nicobarica nicobarica*	*Tupaia nicobarica nicobarica*	Andaman & Nicobar Island	29.03.1977	M
55.	ZSI	20,766	*Tupaia nicobarica nicobarica*	*Tupaia nicobarica nicobarica*	Andaman & Nicobar Island	30.04.1975	F
56.	ZSI	21,307	*Tupaia nicobarica nicobarica*	*Tupaia nicobarica nicobarica*	Andaman & Nicobar Island	30.07.1984	M
57.	ZSI	21,303	*Tupaia nicobarica nicobarica*	*Tupaia nicobarica nicobarica*	Andaman & Nicobar Island	30.06.1984	F
58.	ZSI	21,306	*Tupaia nicobarica nicobarica*	*Tupaia nicobarica nicobarica*	Andaman & Nicobar Island	17.07.1984	M
59.	ZSI	21,299	*Tupaia nicobarica nicobarica*	*Tupaia nicobarica nicobarica*	Andaman & Nicobar Island	14.07.1984	F
60.	ZSI	6535	*Tupaia nicobarica nicobarica*	*Tupaia nicobarica nicobarica*	Andaman & Nicobar Island		M
61.	ZSI	21,305	*Tupaia nicobarica nicobarica*	*Tupaia nicobarica nicobarica*	Andaman & Nicobar Island		M
